# Severe Complications in Testicular Germ Cell Tumors: The Choriocarcinoma Syndrome

**DOI:** 10.3389/fendo.2019.00218

**Published:** 2019-04-12

**Authors:** Katarina Rejlekova, Maria C. Cursano, Ugo De Giorgi, Michal Mego

**Affiliations:** ^1^2nd Department of Oncology, Faculty of Medicine, National Cancer Institute, Comenius University, Bratislava, Slovakia; ^2^Oncology Unit, Università Campus Bio-Medico, Rome, Italy; ^3^Medical Oncology Department, Instituto Scientifico Romagnolo per lo Studio e la Cura dei Tumori, IRCCS, Meldola, Italy

**Keywords:** choriocarcinoma syndrome, testicular germ cell tumor, choriogonadotropin, acute respiratory failure, lung metastases

## Abstract

Testicular germ cell tumors (TGCTs) represent the most common solid tumor in young men and is a model of curable cancer. The effectiveness of cisplatin-based chemotherapy secures more than 95% of patients' 5-years survival rate. However, some high-risk patients with a very advanced disease develop choriocarcinoma syndrome (CS) connected with acute respiratory failure with poor prognosis and high mortality rate shortly after beginning systemic chemotherapy. CS was first described as a syndrome with hemorrhage from metastatic sites in patients with TGCTs with significantly high choriogonadotropin level. Acute hemorrhage to lung metastases is typical, but hemorrhage can occur from any metastatic site. Patognomic of choriocarcinoma cells is an invasion of small blood vessels within CS. The incidence of CS in patients with TGCTs are not well-defined and can vary across the world. To date, there are a few case reports and small retrospective series reporting a connection between systemic chemotherapy and the development of CS in metastatic TGCTs. CS is known to be triggered by massive tumor cell lysis as a result of chemotherapy and cytokine release, aggravated with alveolar hemorrhage. This can lead to a consecutive superinfection, furthered with neutropenia after chemotherapy, acute respiratory distress syndrome, rising to systemic inflammatory response, resulting in multiorgan failure and death. A reasonably effective approach in patients with extensive disease could be a shortened course of chemotherapy as well as a reduction of dosage in induction chemotherapy before full-dose chemotherapeutical regimen; however, current data regarding optimal treatment approach are limited. Patients' referral to tertiary centers and the administration of induction chemotherapy in an intensive care unit setting could further improve the treatment outcome.

## Introduction

Despite the rarity of testicular germ cell tumors, with a count of just 1% from all male malignancies, these tumors represent the most common type of solid tumor in reproductive men between the ages of 20 and 40 years, with an incidence of up to 10 in 100,000 men (1). Due to a unique chemosensitivity to chemotherapy, they represent one of the most curative malignancies overall. More than 95% of patients achieve a 5-years survival rate because of the effectiveness of combined cisplatin-based chemotherapy (2, 3). Germ cell tumors can be classified as either seminomatous or non-seminomatous histologic types, whereby choriocarcinoma is the most aggressive subtype of non-seminomas due to their biologic characteristics (4, 5). International Germ Cell Cancer Collaborative Group (IGCCCG) developed a clinically based prognostic classification for germ cell tumors, which has been used since 1997 (6). The IGCCCG classification stratifies patients into good-, intermediate-, and poor-prognosis subgroups on the basis of three criteria: the primary tumor site, the levels of serum tumor markers, and whether extra-pulmonary visceral metastases are present. The majority of the patients with metastatic disease are allocated in the good-risk group, representing 56% of patients with a non-seminomatous germ cell tumor (NSGCT) with a high curability rate >90%, while patients in the intermediate risk (representing 28% of patients with NSCGTs) and poor prognosis groups (representing 16% of patients with NSCGTs) have 5-years survival rates of only 80 and 48%, respectively. The poor-risk NSGCT is defined by the presence of a mediastinal primary tumor, non-pulmonary visceral metastases, or any of the following serum tumor marker elevations: alpha-fetoprotein (AFP) >10,000 ng/mL, human choriogonadotropin (hCG) >50,000 IU/L and/or lactate dehydrogenase (LDH) >10 times the upper limit of normal rely on IGCCCG classification.

**Figure 1:**
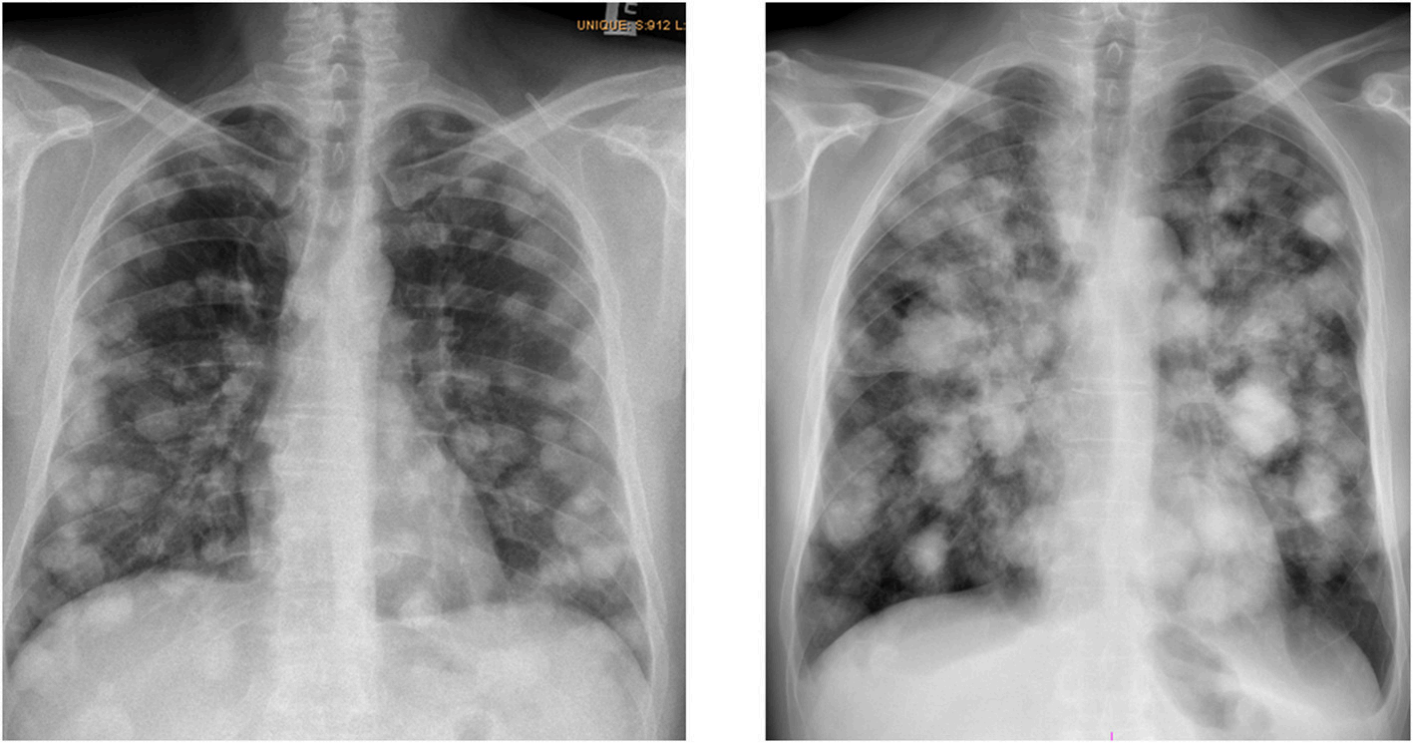
Typical chest X-rays of patients with choriocarcinoma syndrome (patient with multiple lung metastases, histologically proved choriocarcinoma, hCG level 1,600,0000 IU/L, who developed shortly after the administration of the 2-days of EP chemotherapy-choriocarcinoma syndrome), archive of National Cancer Institute, 2017.

The current understanding is that there exists a subgroup of patients with poor-risk NSGCTs, the so-called super-high-risk patients. They are characterized by widespread lung metastases, pure choriocarcinoma, and a high choriogonadotropin level as defined by the European Germ Cell Cancer Collaborative Group EGCCCG (Figure 1) (7, 8). These patients are at a high risk to develop the so-called choriocarcinoma syndrome, which was described for the first time in 1984 by Logothetis et al., as a syndrome with hemorrhage from metastatic sites in patients with advanced germ cell tumors with high-volume of choriocarcinoma elements, especially those with a choriogonadotropin level over 50,000 IU/l (9). Acute hemorrhage to lung metastases is typical, but hemorrhage from any metastatic sites can occur. The patognomic characteristic of choriocarcinoma cells is their invasion to small blood vessels. Typically, choriocarcinoma syndrome occurs shortly after the administration of chemotherapy and is connected with a high risk of fatal bleeding from metastatic lesions and frequently with acute respiratory failure with a high mortality rate at an early phase of the treatment induction (10). Mostly, the hemorrhage appears shortly after the introduction of the chemotherapy, but there are also cases with pretreatment onsets reported in literature (11). The choriocarcinoma syndrome was not only described in pure choriocarcinomas; cases with embryonal tumors or seminomas have also been recorded (12, 13).

The incidence of acute respiratory failure in patients with testicular germ cell tumors (TGCTs) with massive lung involvement and choriocarcinoma syndrome is not well-defined (14). The available literature is missing data on the total percentage of the super-high-risk patient population from the poor-risk group of GCTs, e.g., Moran- Ribon et al. counted 20% from the total of the poor-risk group, but the authors also pointed out the careful interpretation due to selection bias, which could be introduced by the presentation of these patients to a referral center (10).

Until now, there are still controversies on the definition of super-high-risk patients or so-called choriocarcinoma syndrome, as well as the optimal therapeutical approach for this group of patients. Available retrospective data are based on different patients' characteristics, different high-risk features of this patient's group, as well as different primary goals or the study end-points, including different conclusions. Therefore, it is also difficult to determine the incidence of choriocarcinoma syndrome, or ARDS connected with this so-called choriocarcinoma syndrome or mortality rate.

The aim of this article is to describe severe complications in TGCTs related to the choriocarcinoma syndrome, a patognomic unit which deserves a deeper understanding of its etiopathogenesis, clinical manifestation, and the selection of high-risk patients corresponding to better (optimal) therapeutical approach for them, in order to decrease mortality in the early phases of induction chemotherapy without compromising their survival.

## Pathophysiology

The exact pathogenesis of choriocarcinoma syndrome is currently unknown. Choriocarcinoma is the rarest subtype of germ cell malignancy with pure examples representing under 1% of the total number of NSGCT, while 7–8% of testicular tumors contain a choriocarcinoma component (15). Choriocarcinoma is composed of cytotrophoblast, intermediate trophoblast and syncytiotrophoblast cells. Cytotrophoblasts represent a trophoblastic stem cell, whereas the syncytiotrophoblast represents a more terminally differentiated cell. The typical pattern is a plexiform arrangement of syncytiotrophoblast cells with mononucleated, mostly cytotrophoblast cells around foci of hemorrhage, although some examples may have a relatively inconspicuous syncytiotrophoblast component. In developmental embryology, these cell types secrete hCG to promote the maintenance of corpus luteum and they are essential for the implantation and subsequent placentar development (16). Pure or choriocarcinoma-predominant tumors are characterized by high levels of hCG, which may be associated with a different clinical signs and symptoms (15). Choriocarcinoma is characterized by rapid proliferation, invasiveness, vascularity, and a tendency to outgrow its blood supply with subsequent necrosis of tumor (14). Metastases to regional lymph nodes, as well as hematogenous spread to lung, liver and brain, occur at an early stage (5). A propensity for hemorrhage is well-recognized in patients with gestational trophoblastic disease, the female counterpart of testicular choriocarcinoma (17). Although choriocarcinoma in the male is a less common entity, a similar tendency for hemorrhage exists. Hemorrhage has been implicated as the cause of death in 44% of patients with testicular choriocarcinoma at autopsy (4). These processes reflect the biological behavior of choriocarcinoma cells, which directly invade, erode and destroy blood vessels. It is also believed that some products of tumor cells elaborate on the damage of blood vessels without their direct invasion (12). The basic mechanism of choriocarcinoma syndrome is probably massive tumor lysis, resulting from chemotherapy. Subsequent cytokine release, aggravated with alveolar hemorrhage, can lead to acute respiratory failure (ARDS) and death (18). The pathogenesis of ARDS in patients with TGCTs is probably multifactorial and involves massive lung metastases, massive intra-alveolar tumor-lysis, early necrosis of tumor cells, and consecutive superinfection, which can be furthered with neutropenia after chemotherapy. The pathogenesis of intra-alveolar tumor-lysis is probably the same as in the case of tumor-lysis syndrome (TLS), which is characterized by the release of potassium, phosphorus, nucleic acids, and their metabolites, such as ureic acid and cytokines (19). While the occurrence of TLS with hematological malignancies and some aggressive solid tumors is consistently described, such events have not exactly been reported in poor-risk germ cell tumors. Case reports in TGCTs described as an early death due to TLS, could be related to pulmonary distress with multifactorial pathogenesis in super-high-risk patients without metabolic signs of TLS in this group of patients (20). In case of choriocarcinoma syndrome; releasing of the cytokines is probably raising to systemic inflammatory response directing to multiorgan failure (21).

Another factor that could play the additive role in the pathogenesis of choriocarcinoma syndrome is paraneoplastic hyperthyroidism induced by a very high level of choriogonadotropin. In patients with hCG over 50,000 U/l, the incidence of hyperthyroidism could be present in more than 50% of patients (22). hCG is dimeric molecule composed of two subunits, an alpha subunit which is identical with an alpha subunit of the thyroid-stimulating hormone (TSH) and hormone-specific beta subunit. Due to the structural similarity of the alpha subunits TSH and hCG, the stimulation of the thyroid-stimulating hormone- receptor (TSH-r) by hCG can lead to thyrotoxicosis and worsen the patient's condition, as well as the course of acute respiratory failure (23).

## Treatment Outcome

### Treatment Approaches Without a Reduced-Dose Intensity During Induction Chemotherapy

There is no effective preventive precaution against the choriocarcinoma syndrome. To date, there are just few case reports and small retrospective series recording the connection between systemic chemotherapy and the development of the choriocarcinoma syndrome and ARDS in metastatic TGCTs (10, 11, 13, 18, 21, 24).

The first retrospective study by Moran-Ribon et al., published in 1994, involved 11 super-high-risk patients with TGCTs treated between 1982 and 1989 at the Gustave Roussy Institute (10). All these patients developed poor-risk TGCTs after 35 days of chemotherapy administration, and experienced acute respiratory failure with the need of mechanical ventilation (MV). All of them died within the first 5 weeks of chemotherapy, despite their admission to the Intensive Care Unit (ICU) before the beginning of therapy. Eight of them had extensive pulmonary metastases, three patients had hilar lymphadenopathy, and three others had bulky mediastinal adenopathy with atelectasis and pleural effusion. Six patients had hCG over 100,000 IU/L, and in 9 patients there was a presence of hypoxemia PaO_2_ <60 mm Hg (8 kPa) at the time of their admission to ICU. Different cisplatin-based chemotherapeutical protocols were used.

During the period of observation all patients developed fever, and septicemia was documented in 7 of them, with the empiric antibiotic coverage in all of them. In 5 patients, the antibiotics were not active and in 2 patients just one demonstrated some activity, when compared the results of antibiograms of the isolated microorganism, retrospectively. None of the patients had a tumor lysis syndrome. In these patients, there was an increase of alveolar abnormalities and the objective response on the lungs was a minor response in four, a stable disease in four, and progression in three patients. At the time of the patients' death, the lungs became “uniformly white” on chest X-rays. Four (36%) patients had an autopsy, with the results indicating the presence of necrotic and fibrotic tissue but with the viable residual tumor in all of them. Authors hypothesized multifactorial etiology of ARDS in this super-high-risk group of patients and the clinical presentation was compatible to the choriocarcinoma syndrome reported by Logothetis (9).

In 2003, Kirch et al. published a retrospective study of 16 poor-risk patients with TGCTs, who were referred to the ICU for treatment due to respiratory distress and a high risk of complications after the administration of chemotherapy during the 10-years period of time (18). They were identified using a computerized database, based on the heavy tumor burden, disseminated lung metastases, and elevated tumor marker levels. All patients had pulmonary metastases, nine (56%) patients also had extrapulmonary involvement, with a median level of hCG 81,700 IU/l. Most patients were hypoxemic, and all of them had dyspnea at the time of the admission to the ICU. Treatment regimens consisted mainly of either etoposide-cisplatin, or adriamycin, cyclophosphamide and cisplatin, or adriamycin, cyclophosphamide and vincristine. Only 1 patient was treated with the regimen containing bleomycin. Nine patients developed ARDS, requiring mechanical ventilation within 3 days after the initiation of the chemotherapy, while seven patients improved immediately after the administration of induction chemotherapy. On admission to the ICU, the median PaO_2_ under the room air was 46 mm Hg (range 44–63 mmHg) for the patients who required intubation, compared to a median PaO_2_ of 80 mmHg (range 70–109 mmHg) for those who remained stable after the chemotherapy. Six patients also experienced tracheal/bronchial hemorrhage after the course of chemotherapy. Two patients were afflicted with the biological tumor lysis syndrome, as the study reported. Seven ventilated patients were febrile during the observation, while fever was microbiologically documented in five of them. Seven patients experienced chemotherapy-induced aplasia, with a median duration of neutropenia of 10 days. Three (19%) patients developed nosocomial pneumonia and in two of them it was the leading cause of death. Six (86%) patients who did not require MV were discharged alive. Only one patient out of nine requiring MV remained alive, while seven patients died under mechanical ventilation and the last one was weaned off MV but died due to the sepsis arising from a superinfected necrotic tumor on day 41 after chemotherapy administration. Five patients received an autopsy, which indicated that ARDS was the first cause of death in three of them, and pulmonary aspergillosis in one, whereas a massive tumor invasion with hemothorax due to metastases rupture was observed in another autopsied patient. In the study, the development of ARDS was independent of the level of hCG as well as with the applied chemotherapeutical regimen. The only one predictor of the intubation was the initial PaO_2_/FiO_2_ ratio at the time of the admission to the ICU. Refractory hypoxemia and ventilator-associated pneumonia were the leading cause of death. Bleomycin, which toxicity can be aggravated with high oxygen concentration, could not be responsible for the worsening of the respiratory functions because only one patient was actually treated with it.

### Treatment Approaches Utilizing Alternative Regimen During Induction Chemotherapy

Based on this knowledge, new alternative approaches have been applied for this group of patients due to the risk of evolution of the choriocarcinoma syndrome or acute respiratory failure, with the aim to improve their prognosis. The effective approach appeared to be either reduced or shortened course of the induction chemotherapy before the administration of the full-dose chemotherapeutical regimen. Three retrospective studies tested the alternative regimens during induction chemotherapy to decrease the mortality rate due to acute complications connected with administered chemotherapy (24–26)

Massard et al. retrospectively evaluated all patients treated for poor-risk TGCTs and multiple lung metastases at Institut Gustave Roussy from April 1982 to November 2006. A total of 25 patients with extensive lung metastases, dyspnea, at the time of the admission to the specialized intensive care unit or hypoxia (defined as a partial pressure of oxygen PO_2_ <80 mmHg), or both criteria, were selected. Until 1997, a cohort of 15 patients was treated with full-dose cisplatin-based chemotherapy. Since 1997, a second cohort of 10 patients were treated with the reduced regimen of induction chemotherapy. The regimen consisted of 3 days of EP without bleomycin, and the remaining 2 days of chemotherapy were postponed to approximately day 15. After induction chemotherapy, the full-dose regimen with a standard dose of BEP on day 21 was started. This approach lowered the incidence of ARDS after 1997 by 57% (87 vs. 30%), as well as the mortality rate of ARDS by 40% (60 vs. 20%). Long-term survival increased from 27 to 40% (24).

Gillessen et al. retrospectively evaluated 20 patients with metastatic TGCTs (18 patients with poor prognosis), treated in St Bartholomew's Hospital, between 1998 and 2009 (25). Patients were treated with the low-dose induction chemotherapy baby-BOP (bBOP) on day 1 only, with subsequent chemotherapy BEP introduced 7–10 days after the bBOP regimen (25). bBOP was administered to 9 patients because of poor performance status (≥3), due to an extensive retroperitoneal disease in 6 patients, brought about by pulmonary embolism at diagnosis in two patients and hydronephrosis and impaired renal function in four patients. Two patients were presented with the vena cava superior obstruction, and two patients had imminent or present respiratory failure and serious gastrointestinal bleeding due to stomach metastasis. bBOP was well-tolerated, without any toxic deaths, or neutropenic sepsis. In one patient with a massive pulmonary disease, tumor-related death occurred during the first cycle, due to cystic transformation growth, resulting in pulmonary insufficiency and death, while the level of tumor markers decreased. Response to bBOP was not inferior to the standard first-line BEP regimen in this group of poor-risk patients. The 2-year PFS and OS rates for 18 poor-risk patients with induction bBOP were 72 and 79%, respectively, which were not significantly different compared to poor-risk patients treated without induction bBOP in their institution, with 2-year PFS and OS rates at the level of 75 and 80%, respectively (25).

In the study published by Tryakin et al. in 2018, authors retrospectively assessed 63 (24%) out of 265 patients with poor risk metastatic GCTs, all with ultra-high tumor marker levels and/or an ECOG performance status of 3–4 (26). Before 2005, these patients were treated with full dose BEP; after 2005, the patients were treated with abbreviated EP as the induction chemotherapy, followed by subsequent full-dose chemotherapy. Of these 63 patients, 50 (79%) had hCG ≥200,000 IU/L, 50 (79%) had lung metastases, and 50 (79%) had liver metastases, respectively. Respiratory insufficiency was observed in 28 of the patients; 22 patients had multiple pulmonary metastases with a high level of hCG, two patients presented with pulmonary embolism, while four had a massive mediastinal tumor at the time of the diagnosis. Hemoptysis was present in 26 patients with multiple lung metastases and 20 complained of grade ≥3 pain. Forty-five (71%) out of 63 patients were treated will full doses of cisplatin- and etoposide-based chemotherapy during the first cycle, while 18 (29%) received a reduced cycle of EP as induction chemotherapy. Patients who received the first cycle of reduced chemotherapy EP developed fewer acute life-threatening toxicities, compared to patients treated with the full-dose first cycle of chemotherapy (44 vs. 76%, *P* = 0.01). The rates of severe hematological toxicities for the reduced- compared to full-dose chemotherapy group (grade 4, 56% vs. 96%, *p* = 0.0004) and infectious complications (grade 3–4, 28 vs. 53%, *p* = 0.09) were lower. The 5-years OS rates was 52%, equal in both groups (HR 0.99, 95% CI 0.44–2.26, *p* = 0.99). However, OS in the group of patients with ultra-high tumor marker levels (*n* = 63), compared with the group of other patients with poor-risk (*n* = 202), did not differ significantly (HR 0.89, 95% CI 0.58–1.36, *P* = 0.59 (25, 26).

To date, there is no exact feature associated with the higher risk of the complications after the administration of the chemotherapy within this group of patients. It seems to be reasonable that massive lung involvement is the main risk factor in the pathogenesis of ARDS. But it is also possible that chemotherapy can be associated with the worsening of the respiratory functions, as the studies by Moran Ribon et al. and Kirch et al. concluded (10, 18). On the other hand, the evolution of ARDS in the Kirch et al. study was independent of the known prognostic factors as a histologic type of the tumor, stage of the disease, tumor marker levels, or given chemotherapeutical regimen (18). The only predictor of the need of MV was the initial PaO_2_/FiO_2_ ratio before chemotherapy administration. At the same time, tumor-associated ARDS can be explained as a consequence of administered chemotherapy, because the peak of tumor markers was observed 2 days after chemotherapy administration (18). Based on this knowledge, a reasonably effective approach can be a reduced respectively, shortened course of induction chemotherapy before the administration of consecutive full-dose chemotherapy as the eventual prevention of ARDS development in these patients. All three retrospective studies with modified induction chemotherapy showed non-inferior treatment results compared to full-dose induction chemotherapy (24–26). These studies suggest that reduced induction chemotherapy can be helpful to decrease the incidence of ARDS in this group of patients. However, the significant improvement of the reduction on the mortality of ARDS could probably be multifactorial and can be connected to improved supportive care and the preventive administration of the granulocyte-colony stimulating factor (G-CSF). The acute tumor and /or alveolar hemorrhage was present in more than 30% of the patients in all mentioned studies, which could eventually aggravate respiratory failure. Secondary infection in neutropenic patients, as well as interstitial lung fibrosis, can be the leading cause of consecutive ARDS and may be an associated cause of death.

In the Moran Ribon et al. and Kirsch et al. studies, neutropenia occurred in almost half of the patients; septicemia was also observed and described (10, 18). At the same, we need to realize that ventilator-associated pneumonia is hard to diagnose. Lung parenchyma is characterized by local immunosuppression because of necrotic tumor masses, which allow bacterial colonization and consequent infection. An early diagnosis of ventilator-associated pneumonia and consequent adequate antibiotic therapy is crucial in this situation. Bronchoscopy with bronchio-alveolar washing and bronchial brushing may allow the isolation of adequate bacteriological samples, as well as the removal of blood clots. Immunosuppression after chemotherapy can be an independent risk factor associated with a higher mortality rate of these super-high-risk patients with relative risk at 2.3% (27).

We need to realize that all mentioned studies had their limitations due to retrospective character, and different time frames of treatment with full-dose and reduced-dose chemotherapy, which could account for selection bias and at the same time, could also participate in better treatment results with reduced-dose chemotherapy. The biggest deficiency of all discussed studies was a low number and high heterogeneity of the patients (Table 1).

**Table 1:** Summary of studies of super high-risk patients.

**References**	**Number of patients**	**Level of hCG IU/L**	**Pulmonary mts**	**Other mts**	**Initial pO2 mmHg**	**Full dose/reduced dose chemotherapy**	**Chemotherapeutical regimen**	**Infection complications no pts**.	**ARDS/death from ARDS**	**Hemorhage no pts**.	**Death due to ARDS/survival**
Moran-Ribon et al. (10)	11	(9,114–1,080,000) median >100,000	8 (73%)	Mediastinal LAP, hilar LAP, kidney, liver, pancreas, adrenal gland	<60 mmHg 9(82%) >60 mmHg 2 (18%)	11 (100%)–full dose	Different cisplatin-based regimens	7 (64%) 11 (100%) febrile	11(100%)	NR	All died
Kirch et al. (18)	16	(33–413,000) median 81,700	9 (56%)	Liver, bones, brain	<70 mmHg 16(100%) <46 mmHg 9(56%)	16 (100%)–full dose	EP, adriamycin, cyclophosphamide, or adriamycin, cyclophosphamide, vincristine	5 (31%) 7 (44%) febrile	9(56%)	6(38%)	8/9 (89%) (on MV) died 1/7 (14%) (without MV) died
Massard et al. (24)	25	(11–8,920,000) median >200,000	25 (100%)	NR	<80 mmHg 2(8%)	15 (60%)–full dose 10 (40%)–reduced EP	Cisplatin-based regimens-full dose EP-reduced	NR	13 (87%_–full dose 3 (30%)–reduced dose	NR	9/15 (60%) died–full dose 2/10 (20%) died –reduced dose
Gillessen et al. (25)	20	(1–1,250,000) median 35,195	NS, 1 (5%)-massive	RP LAP, stomach, brain, bones	NR	20 (100%)–reduced-bBOP	bBOP, BEP	NR-no toxic death, no neutropenic sepsis after bBOP	None	2 (10%)	None died due to ARDS
Tryakin et al. (26)	63	(0–20,525,000) median >200,000	50 (79%)	Liver, mediastinal LAP, RP LAP, brain	NR	45 (71%)–full dose 18 (29%)–reduced dose	BEP, EP	Gr.3,4 24 (53%)-full dose vs. 5 (28%)	NR	NR	NR

*NR, non-reported; RP LAP, retroperitoneal lymphadenopathy; BEP, bleomycin, etoposide, cisplatin; EP, etoposide, cisplatin; ARDS, acute respiratory distress syndrome; LAP, lymphadenopathy; bBOP, baby BOP-bleomycin, vincristine, cisplatin; MV, mechanical ventilation*.

In the review by Reilley, the author brought a comprehensive view over molecular pathology, clinical features of choriocarcinoma, as well as diagnostic and therapeutic challenges in this unique but aggressive germ cell malignancy due to complications arising from an underlying disease and its treatment. He pointed out the importance of treatment individualization based on the extent of the disease, e.g., for the patient with high-volume lung metastases, etoposide and cisplatin without bleomycin as induction regimen, should be administered to avoid fatal respiratory failure (28).

## Conclusion and Future Direction

Choriocarcinoma syndrome is a rare but life-threatening condition in “super high-risk” patients with testicular germ cell tumors that are characterized with widespread lung metastases, choriocarcinoma histology, and a high choriogonadotropin level. Such patients are often ineligible for prospective trials. Their eventual inclusion in reports as early deaths is recommended when interpreting the results of clinical trials in patients with poor-risk TGCTs. The optimal therapeutic approach for this group of patients remains to be defined. Current evidence suggests that the best treatment results in these “superhigh-risk” patients are obtained in high-volume reference centers (1). Because of the rarity and complexity of this super-high-risk group of patients, there is imperative need for their early referral to specialized centers (if possible within 24 h) to optimize their chances of survival, as indicated by retrospective data and by the recommendations of TGCT guidelines (7, 8). According to EGCCCG, patients with widespread lung metastases, pure choriocarcinoma, and high hCG should be treated by 2–3 days of full-dose cisplatin and etoposide with the continuation of chemotherapy after the patient′s recovery (7, 8). Bleomycin should be avoided during induction chemotherapy since it may induce pulmonary fibrosis. However, it should be re-introduced once the patient's condition allows that, because its complete omission was demonstrated to be detrimental in a prospective randomized trial (7, 8).

Prospective translational trials that will lead to a better understanding of the pathophysiology of the choriocarcinoma syndrome and development of new biomarkers for better stratification of super-high-risk patients are warranted. Due to its low prevalence, international collaboration and clinical trials utilizing new treatment strategies are needed to decrease the mortality rate caused by this rare but highly fatal condition.

## Author Contributions

KR, MC, UD, and MM participated in the conception and design of this study. MM and KR drafted the article and all authors reviewed it critically for its important intellectual content.

### Conflict of Interest Statement

The authors declare that the research was conducted in the absence of any commercial or financial relationships that could be construed as a potential conflict of interest.
